# Translation, Cross-Cultural Adaptation, and Validation of the Malay Version of the System Usability Scale Questionnaire for the Assessment of Mobile Apps

**DOI:** 10.2196/10308

**Published:** 2018-05-14

**Authors:** Muhamad Fadhil Mohamad Marzuki, Nor Azwany Yaacob, Najib Majdi Yaacob

**Affiliations:** ^1^ Department of Community Medicine School of Medical Sciences Universiti Sains Malaysia Kelantan Malaysia; ^2^ Unit of Biostatistics and Research Methodology School of Medical Sciences Universiti Sains Malaysia Kelantan Malaysia

**Keywords:** usability, System Usability Scale, Malay, questionnaire translation, questionnaire validation, mobile app

## Abstract

**Background:**

A mobile app is a programmed system designed to be used by a target user on a mobile device. The usability of such a system refers not only to the extent to which product can be used to achieve the task that it was designed for, but also its effectiveness and efficiency, as well as user satisfaction. The System Usability Scale is one of the most commonly used questionnaires used to assess the usability of a system. The original 10-item version of System Usability Scale was developed in English and thus needs to be adapted into local languages to assess the usability of a mobile apps developed in other languages.

**Objective:**

The aim of this study is to translate and validate (with cross-cultural adaptation) the English System Usability Scale questionnaire into Malay, the main language spoken in Malaysia. The development of a translated version will allow the usability of mobile apps to be assessed in Malay.

**Methods:**

Forward and backward translation of the questionnaire was conducted by groups of Malay native speakers who spoke English as their second language. The final version was obtained after reconciliation and cross-cultural adaptation. The content of the Malay System Usability Scale questionnaire for mobile apps was validated by 10 experts in mobile app development. The efficacy of the questionnaire was further probed by testing the face validity on 10 mobile phone users, followed by reliability testing involving 54 mobile phone users.

**Results:**

The content validity index was determined to be 0.91, indicating good relevancy of the 10 items used to assess the usability of a mobile app. Calculation of the face validity index resulted in a value of 0.94, therefore indicating that the questionnaire was easily understood by the users. Reliability testing showed a Cronbach alpha value of .85 (95% CI 0.79-0.91) indicating that the translated System Usability Scale questionnaire is a reliable tool for the assessment of usability of a mobile app.

**Conclusions:**

The Malay System Usability Scale questionnaire is a valid and reliable tool to assess the usability of mobile app in Malaysia.

## Introduction

The advancement of communication technologies has changed the way people search for and find information. This is especially prevalent in the case of health-related information. Consequently, health providers should update their health education and promotion strategies to disseminate information from conventional printed material such as pamphlets and flip charts, to more interactive and updated material such as mobile apps [1]. Mobile apps have the advantage of being widely available soon after development through multiple platforms. The usability of the mobile app in question plays an important role in determining its effectiveness to improve health knowledge and awareness. An app must not only be user-friendly, but it should also attract users.

Usability is defined as the extent to which a product can be used by specified users to achieve specific goals effectively and efficiently as well as providing user satisfaction in a specified context of use [2]. Questionnaire surveys are among the established and acceptable methods for system usability evaluation [3]. Usability consists of 5 quality attributes of the system which assess how easy user interfaces are to use [4], namely learnability, efficiency, memorability, system errors, and user satisfaction.

Generally, there are two methods of assessing the usability of a product, expert reviews and usability testing [5]. Many questionnaires have been developed for usability assessment of computer-based interfaces, websites, apps, or any software or hardware with which users interact. These include the After Scenario Questionnaire (ASQ), Computer System Usability Questionnaire (CSUQ), and the Usefulness, Satisfaction and Ease of Use (USE) questionnaire [6]. The usability questionnaires recommended for the assessment of mobile apps can range from two simple post-test questions, to standard questionnaires such as the Post-Study Usability Questionnaire (PSSUQ) or the System Usability Scale (SUS) [7,8].

The System Usability Scale (SUS) is one the most widely used questionnaires to assess the usability of a system or product [9]. It was developed by John Broke in 1986 in response to the demand of many industries for a simple, quick, and cost-effective method to assess the usability of a system [10]. It has been utilized in various surveys to determine the usability of wide range of user interfaces such as standard operating system-based software interfaces, Web-pages, mobile apps, and networking equipment [6]. Originally, the SUS was developed for Digital Equipment Co Ltd customers who are the native English speakers [9]. The SUS questionnaire has since been translated into many languages including Spanish, French, Dutch, Portuguese, Slovenian, Persian, German, and more recently Indonesian. All translated versions have shown similar internal reliability to the original English version [11].

To the best of the authors’ knowledge, there are no studies reporting the translation of the SUS questionnaire into Malay, despite the widespread usage of this questionnaire across the world. It is crucial to have a SUS questionnaire in the local language to accurately capture the thoughts, feelings, perceptions, behaviors, and attitudes of local users towards the usability of the tested product. Different cultures can interpret similar words or phrases in a different manner, therefore the translation used in this study takes into consideration the linguistics of the questionnaire, as well as the cross-cultural adaptation needed to maintain the validity of the questionnaire [11]. Thus, the objective of this study is to translate and validate the original English version of the SUS into Malay.

## Methods

### Overview

The SUS was developed by John Brooke in 1986 [10] and consists of a 10-item questionnaire scored on a 5-point Likert scale from 0 (strongly disagree) to 5 (strongly agree). The questionnaire is arranged to alternate between positive and negative statements to avoid habitual bias from the respondent. The score contribution for the odd items (the positive statements) is the scale position minus 1 and the contribution for the even items (the negative statements) is 5 minus the scale position. The overall score is calculated from a sum of all item scores multiplied by 2.5 and can range from 0 to 100. A system or product that received score of 68 and above is considered to have good usability [10].

### Adaptation Process

The original SUS questionnaire was translated into Malay using international guidelines for cross-cultural adaptation to ensure the quality of the translated version and its consistency of meaning to the original version [12]. First, the forward translation process (from English to Malay) was conducted by two translators and a report of the translation was produced by both translators. The two translations were synthesized into one document after a thorough discussion which addressed any gaps or differences between the two reports.

The original and translated versions of the SUS questionnaire were given to two groups of native Malay speakers who spoke English as their second language. Each group consisted of 8 translators who received either the original or translated questionnaire version and then performed either the forward or backward translation respectively. The forward and backward translation discrepancies were reconciled, and cross-cultural adaptation was done to derive the final version. Since the purpose of translating the SUS questionnaire is to assess the usability of mobile apps, the word “system” has been changed to “mobile application” in the survey. The Malay term for this is “aplikasi mudah alih,” hence the adapted questionnaire is called *Skala Kebolehgunaan Aplikasi Mudah Alih* (SKAMA) in Malay.

### Validation Process

The SKAMA questionnaire was subsequently validated in terms of its content validity, face validity, and reliability (internal consistency). Content validation aims to assess the relevancy and representativeness of each item to a specific domain by a panel of experts. In this context, it will assess the relevance of all 10 items in the SKAMA to represent the usability domain.

**Figure 1:**
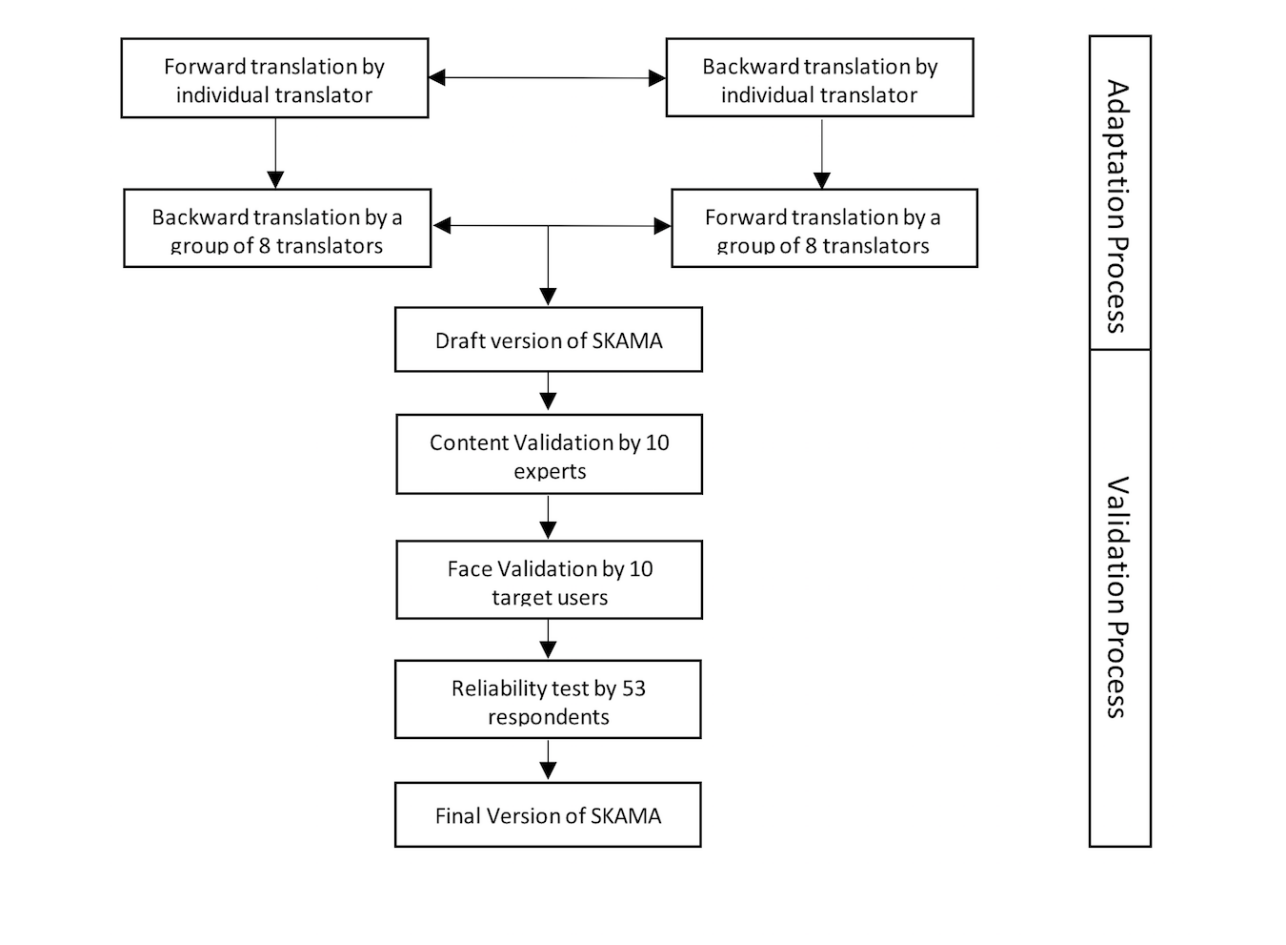
Flowchart of the validation process.

Content validation of the SKAMA questionnaire was conducted by 10 experts (including 2 mobile app developers) who were asked to give a score of 1 (item not relevant) to 4 (item very relevant), based on the relevancy of the translated items in the SKAMA, to assess the usability of a mobile app. Scores of 3 and 4 were recategorized as 1 (relevant) and scores of 1 and 2 as 0 (not relevant). The content validity index (CVI) was computed by calculating the scale average [13]. Figure 1 gives an overview of the validation process.

Face validation testing, which aims to assess the clarity and comprehensibility of the translated items, was conducted by 10 target users. The users were asked to give score from 1 (item not clear and not understandable) to 4 (item very clear and understandable) based on the clarity and comprehensibility of the translated items in the SKAMA questionnaire. Scores of 3 and 4 were recategorized as 1 (clear and understandable) and scores of 1 and 2 as 0 (not clear and understandable). The face validity index (FVI) was computed by calculating the scale average [13]. Reliability testing was conducted on 49 respondents based on a minimum sample size estimation to assess the internal consistency [14]. They were asked to use the SKAMA to assess the usability of the Facebook mobile app on their mobile phone. The reliability analysis was computed using R software. All three validation tests performed on the SKAMA questionnaire were conducted using an online Google Form where the link was sent to each respondent via a personal WhatsApp (for the validation test) or a group WhatsApp (for the reliability test) to facilitate the data collection.

This study has been approved by the National Medical Research Registry, Malaysia [NMRR-17-2623-38675 (IIR)] and Human Research Ethics Committee USM, Malaysia (USM/JEPeM/17110601).

## Results

In the translation of the SUS questionnaire, the word “system” was changed to the Malay word for “mobile application,” namely “*aplikasi mudah alih,”* as the Malay adaptation of the SUS questionnaire is intended to determine the usability of mobile apps. The CVI (Table 1) and FVI (Table 2) of SKAMA were calculated to be 0.91 and 0.94 respectively. The CVI and FVI score of above 0.83 for both tests indicates that all items in the questionnaire are relevant to the domain, clear, and comprehensible for the target users [13,15].

The reliability testing was conducted using 53 target users (the minimum estimated sample size was 49 respondents) who responded to the online questionnaire via a URL link sent to them. The age of the respondents ranged from 23 to 60 years. The majority of the target users worked for the government and have a tertiary education. Table 3 shows the characteristics of the target users who responded to the online questionnaire.

**Table 1:** Content validity index based on the rating of the relevancy of items by 10 experts.

Item	E^a^1	E	E3	E4	E5	E6	E7	E8	E9	E 10		I-CVI^b^
Q1	4	4	4	3	4	4	4	4	3	4		1.00
Q2	2	3	4	3	4	3	4	4	4	1		0.80
Q3	4	4	2	4	4	4	4	4	4	4		0.90
Q4	4	4	4	4	3	4	4	4	4	1		0.90
Q5	3	4	3	4	4	4	4	1	4	4		0.90
Q6	4	4	4	4	4	4	4	4	4	4		1.00
Q7	4	3	4	4	3	4	3	3	4	4		1.00
Q8	4	1	3	4	3	3	4	4	1	3		0.80
Q9	3	3	3	4	3	4	4	4	4	4		1.00
Q10	4	1	4	4	3	2	4	3	4	3		0.80
Content validity index average											0.91	

^a^E: Expert.

^b^i-CVI: Item Content Validity Index.

**Table 2:** Face validity index based on the rating of the clarity and comprehensibility of items by 10 target users.

Item	R^a^1	R2	R3	R4	R5	R6	R7	R8	R9	R 10		I-FVI^b^
Q1	3	4	4	4	4	4	4	2	4	4		0.90
Q2	3	4	4	3	4	4	4	3	4	4		1.00
Q3	4	4	4	4	4	4	4	3	4	4		1.00
Q4	4	4	4	4	4	4	4	3	4	4		1.00
Q5	3	4	3	4	4	4	4	1	4	4		0.90
Q6	2	3	4	4	3	4	4	1	3	3		0.80
Q7	3	3	4	3	4	4	4	2	3	4		0.90
Q8	4	4	4	4	3	4	4	2	4	4		0.90
Q9	3	4	4	4	4	4	4	3	4	4		1.00
Q10	3	3	4	3	4	4	4	3	4	4		1.00
Face validity index average											0.94	

^a^R: Rater.

^b^I-FVI: Item Face Validity Index.

**Table 3:** Characteristics of target users (N=53).

Characteristic		Value
Age (years), mean (SD)		39.4 (10.46)
**Highest education, n (%)**		
	Primary School	1 (1.9)
	Secondary School	7 (13.2)
	Tertiary education	45 (84.9)
**Occupation, n (%)**		
	Government	38 (71.7)
	Private	6 (11.3)
	Pensioner	3 (5.7)
	Unemployed	6 (11.3)

**Table 4:** The internal consistency of the total-item statistics.

Item	Scale mean if item deleted	Scale variance if item deleted	Corrected item total correlation	Cronbach alpha if item deleted
Q1	35.94	25.478	0.416	.85
Q2	35.70	26.830	0.460	.84
Q3	35.38	26.816	0.674	.83
Q4	35.51	27.370	0.459	.84
Q5	36.02	24.134	0.651	.82
Q6	36.40	24.205	0.653	.82
Q7	35.79	26.475	0.469	.84
Q8	35.79	25.245	0.637	.83
Q9	35.75	22.881	0.793	.81
Q10	36.19	24.887	0.429	.85

The Cronbach alpha for the SKAMA questionnaire was determined to be .85 (95% CI 0.79-0.91) which is similar to the original English SUS questionnaire [10]. A higher alpha value indicates a higher internal reliability of the questionnaire and value more than .70 is acceptable as satisfactory internal reliability [16]. The Cronbach alpha for the questionnaire if an item is deleted (from the questionnaire) also remains consistent without significant difference (Table 4) indicating good internal reliability of the developed questionnaire.

## Discussion

The concept of system usability was first coined in the 1980s, in the field of human-computer interaction, when the first personal computer was developed [17]. Usability is the quality attributes of a system which assess how easy a system interface is to use [4]. These attributes include:

1. The learnability of the system (ie, how well users can learn and use a product to achieve the intended goals [18]).
2. The efficiency of the system (ie, how quickly users can perform the task once they learn the design).
3. The memorability of the system (ie, how easily the user can re-establish proficiency when they return to the system after a period of not using it).
4. The errors from using the system.
5. User satisfaction when using the system.

Ideally the usability evaluation of a system should be considered in every step of prototype development, a process which consists of iterative cycles of prototyping, design, and validation [19]. The usability of a developed system can be evaluated either by expert reviews or by usability testing [5]. Expert reviews can be conducted using heuristic checklists, cognitive walkthrough, and guidelines. This is dependent on the experts’ knowledge and experience and therefore this may not reflect the users’ perception of product usability. On the other hand, questionnaires are specifically developed to explore a construct that cannot be measured directly, such as attitude and practice, as well as the usability of a system. Creating a new questionnaire requires a concerted effort from team members, additional costs, and is time consuming. Therefore, researchers are recommended to adapt established, appropriate, and available questionnaires with documented validity in other languages. Literal translation, however, is not sufficient to produce an equivalent questionnaire. The questionnaire must have a good linguistic translation and must be adapted for cultural differences to maintain the content validity [11]. This is referred to as the cross-cultural adaptation of a questionnaire [20]. Validation, on the other hand, aims to ensure that the translated version questionnaire has the same equivalent properties for measuring the construct as the original version. Cross-cultural adaptation to ensure the integrity of the questionnaire is retained, as translation can be problematic, especially when the two languages have nonequivalent words. It is especially important to take into account the fact that different cultures may interpret similar words or phrases in the questionnaire differently and therefore the intended meaning of the items in the questionnaire could be altered from the original version.

Malay is the native language in Malaysia, although multi-ethnic groups do exist. It is for this reason that this study aimed to translate the SUS questionnaire into Malay for use in Malaysia. The Malay version of the SUS, SKAMA, was reviewed by experts in the field, which included mobile app developers, as the aim is to use this translation for the assessment of mobile apps. Therefore, in the translation, the word “system” in the original SUS was replaced with the Malay term for “mobile application.” The experts reviewed the SKAMA questionnaire content in relation to assessment of the usability of mobile apps, taking into account the considerations of local users. Face validity testing tested the clarity of the items to assess usability of mobile app from the target user point of view. Developers and experts in mobile apps may have a different view of system usability compared to the public users, who are the target users when new apps are developed. These two different groups of reviews help to ensure content coverage, while taking into consideration the comprehensibility of the items in the questionnaire to the target user. The high CVI and FVI of SKAMA thus indicates the content is well adopted into local context and translated using clear and understandable sentences.

The reliability of a questionnaire contributes to the validity of it and measures the stability of the questionnaire in terms of consistency of the response. Internal consistency is one of the reliability components used to measure the extent of which the items are measuring the same thing. The most common estimation of internal consistency is the Cronbach alpha coefficient [21]. The high Cronbach alpha value in this study indicates that SKAMA is a reliable tool to assess the usability of a mobile app. The consistent item statistics indicates that all 10 items are measuring a same domain, which is the usability of mobile app. Thus, the SKAMA questionnaire has equal reliability with similar Cronbach alpha values to the original SUS questionnaire and slightly higher values compared to the Indonesian version of SUS [9,11].

In conclusion, the SKAMA questionnaire is a valid tool to measure the usability of a mobile app for a Malay speaking population. SKAMA may also be used to assess other systems’ usability by rephrasing the word “mobile application” back into “system” as in the original SUS.

## Appendix

Multimedia Appendix 1 Actual questionnaire Skala Kebolehgunaan Aplikasi Mudah Alih (SKAMA) or Mobile Application Usability Scale.

## Abbreviations

CVI: content validity index
FVI: face validity index
SKAMA: Skala Kebolehgunaan Aplikasi Mudah Alih
SUS: System Usability Scale
