# Supplementary material for: Translation, Cross-Cultural Adaptation, and Validation of the Malay Version of the System Usability Scale Questionnaire for the Assessment of Mobile Apps
Source: JMIR Hum Factors. 2018 May 14;5(2):e10308. doi: 10.2196/10308 (PMC5972216; doi:10.2196/10308)
Supplement: Multimedia Appendix 1 [file humanfactors_v5i2e10308_app1.pdf]

<u>Soal Selidik Skala Kebolehgunaan Aplikasi Mudah Alih</u>

Skala Kebolehgunaan Aplikasi Mudah Alih
(SKAMA©)

Bagi soalan di bawah, sila tandakan jawapan yang paling sesuai berdasarkan skala seperti berikut.

Sangat tidak bersetuju	1	2	3	4	5	Sangat bersetuju

	1	2	3	4	5
1. Saya rasa saya ingin kerap menggunakan aplikasi mudah alih ini.					
2. Saya rasa aplikasi mudah alih ini sesuatu yang rumit.					
3. Saya rasa aplikasi mudah alih ini mudah untuk digunakan.					
4. Saya memerlukan bantuan daripada orang lain untuk menggunakan aplikasi mudah alih ini.					
5. Saya mendapati fungsi-fungsi dalam aplikasi mudah alih ini saling bersepadu (berhubungkait) dengan baik.					
6. Saya rasa terdapat banyak kandungan di dalam aplikasi mudah alih ini yang tidak konsisten antara satu sama lain.					
7. Saya membayangkan bahawa kebanyakan orang akan cepat belajar menggunakan aplikasi mudah alih ini.					
8. Saya mendapati aplikasi mudah alih ini leceh untuk digunakan.					
9. Saya berasa yakin menggunakan aplikasi mudah alih ini.					
10. Saya perlu belajar terlalu banyak perkara sebelum boleh menggunakan aplikasi mudah alih ini.					

Hakcipta Universiti Sains Malaysia Terpelihara
(LY2018001684)
